# The Expression of FOXO3a as a Forensic Diagnostic Tool in Cases of Traumatic Brain Injury: An Immunohistochemical Study

**DOI:** 10.3390/ijms24032584

**Published:** 2023-01-30

**Authors:** Aniello Maiese, Federica Spina, Giacomo Visi, Fabio Del Duca, Alessandra De Matteis, Raffaele La Russa, Marco Di Paolo, Paola Frati, Vittorio Fineschi

**Affiliations:** 1 Department of Surgical, Medical and Molecular Pathology and Critical Care Medicine, Section of Legal Medicine, University of Pisa, 56126 Pisa, Italy; 2 Department of Anatomical, Histological, Forensic and Orthopedical Sciences, Sapienza University of Rome, Viale Regina Elena 336, 00161 Rome, Italy; 3 Department of Clinical and Experimental Medicine, University of Foggia, 71122 Foggia, Italy

**Keywords:** traumatic brain injury, FOXO3a, immunohistochemical

## Abstract

Traumatic brain injury (TBI) is one of the most well-known causes of neurological impairment and disability in the world. The Forkhead Box class O (FOXO) 3a is a transcription factor that is involved in different molecular processes, such as cell apoptosis regulation, neuroinflammation and the response to oxidative stress. This study is the first to evaluate the post-mortem immunohistochemical (IHC) positivity of FOXO3a expression in human cases of TBI deaths. The autopsy databases of the Legal Medicine and Forensic Institutes of the “Sapienza” University of Roma and the University of Pisa were retrospectively reviewed. After analyzing autopsy reports, 15 cases of TBI deaths were selected as the study group, while the other 15 cases were chosen among non-traumatic brain deaths as the control group. Decomposed bodies and those with initial signs of putrefaction were excluded. Routine histopathological studies were performed using hematoxylin–eosin (H&E) staining. Furthermore, an IHC investigation on cerebral samples was performed. To evaluate FOXO3a expression, anti-FOXO3a antibodies (GTX100277) were utilized. Concerning the IHC analysis, all 15 samples of TBI cases showed positivity for FOXO3a in the cerebral parenchyma. All control cerebral specimens showed FOXO3a negativity. In addition, the longer the survival time, the greater the positivity to the reaction with FOXO3a was. This study shows the important role of FOXO3a in neuronal autophagy and apoptosis regulation and suggests FOXO3a as a possible potential pharmacological target.

## 1. Introduction

Traumatic brain injury (TBI) is defined as the damage deriving from the application of an external force to the head with alteration in brain function, or other evidence of brain pathology [1,2]. According to 2020 Centers for Disease Control and Prevention (CDC) data, approximately 64.362 TBI-related deaths have occurred in the United States and approximately 176 deaths occur every day [3]. Clinical symptoms of brain injury can be delayed or even absent, and the diagnosis can include imaging or laboratory investigations. There are various complex pathological mechanisms underlying brain damage that occurs following TBI, and recently they have been widely studied using experimental murine models.

Following head trauma, two types of damage develop. Primary damage occurs immediately and is directly caused by mechanical force. Secondary damage develops in the following days or weeks and is related to ischemia and consequent processes, such as oxidative stress, neuroinflammation and apoptosis [4,5]. Forkhead Box class O (FOXOs) are transcription factors that regulate proliferation, differentiation, metabolism, oxidative stress and cellular longevity [6]. They are present in all mammals and are divided into different isoforms expressed in various body tissues. In particular, the FOXO3a is most frequently expressed in the brain, especially in the hippocampus, cerebral cortex, and cerebellum.

In mature neuronal cells, FOXOs play a regulatory role in reactive oxygen species (ROS) pathways, neuroinflammation, apoptosis and autophagy in neurodegenerative diseases [7,8]. Recent studies show that FOXO3a carries out neuroprotective effects against ischemic lesions [9] and is significantly elevated in TBI cases, which are associated with the degree of severity of brain injury [10,11].

Our work is the first experimental study on the immunohistochemical (IHC) positivity of FOXO3a expression in human post-TBI brain tissues. The significant results suggest FOXO3a be a forensic diagnostic tool in the diagnosis of TBI, and a possible therapeutic target to prevent secondary brain injuries.

## 2. Results

**Figure 1:**
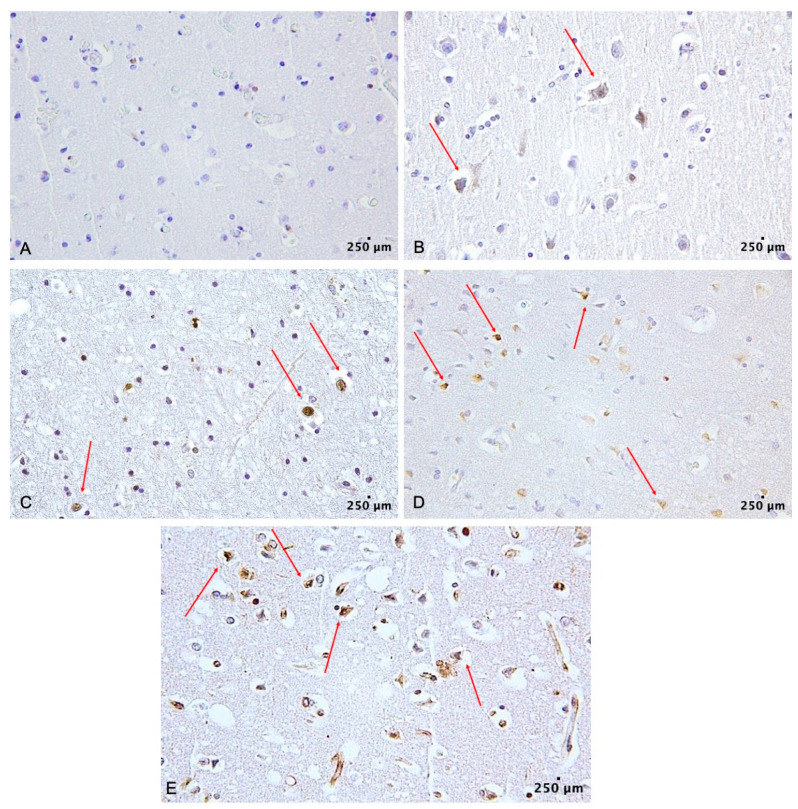
FOXO3a expression in the analyzed cerebral samples (red arrows). The scale bar showed 250 μm in distance. (**A**) FOXO3a is negative (intensity score 0) (100×), (**B**) FOXO3a is positive (Intensity score 1) (100×), (**C**) FOXO3a is positive (intensity score 2) (100×), (**D**) FOXO3a is positive (intensity score 3) (100×), (**E**) FOXO3a is positive (intensity score 4) (100×).

Histologic examination of the cerebral samples showed cerebral hemorrhage (intraparenchymal and subarachnoid). Concerning the IHC analysis, all the 15 samples of TBI cases showed a positivity of FOXO3a in the neuronal cells in the cerebral parenchyma adjacent to the injury (average value of intensity 2.53, *p*-value < 0.05), as shown in Figure 1.

All control cerebral specimens, obtained from the subjects who died of non-traumatic brain death, showed FOXO3a negativity in the neuronal cells (Figure 1A, intensity score 0).

Positivity was greater in subjects who had survived a few hours (Table 1). The longer the survival time, the greater the positivity to the reaction with FOXO3a was.

Statistical analysis via Student’s t-test showed a statistically significant FOXO3a expression for traumatic cases compared to post-mortem injuries and uninjured skin specimens (*p*-value < 0.05).

## 3. Discussion

The results of the study show that in subjects who suffered a TBI, the expression of FOXO3a at the level of the injured brain tissue was significantly increased compared to the control group, which was made up of subjects who died of non-traumatic causes.

**Figure 2:**
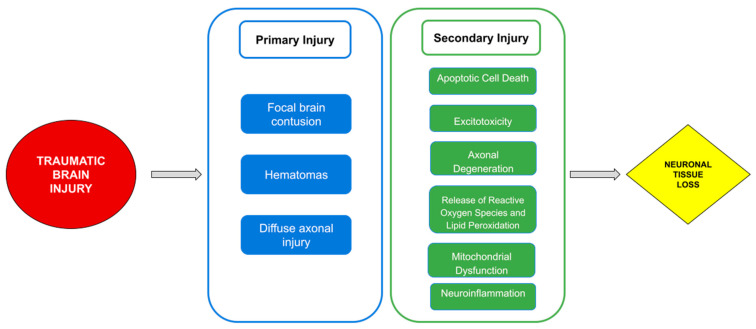
This figure shows the pathophysiological mechanisms of primary and secondary injury after TBI.

The pathogenesis of post-TBI [12] (Figure 2) can be didactically divided into primary brain injury and secondary brain injury.

The former is directly related to the damage exerted by the external force (direct impact, rapid acceleration/deceleration, penetrating injuries and shockwave) during the initial insult. The latter is due to further damages related to the subsequent cellular inflammatory response that occurs following the primary insult.

This distinction is purely didactic as the two injuries often coexist. However, this division allows specific TBI management protocols to be implemented based on which of the two categories the injury falls in.

While the treatment of primary lesions is often surgical [13,14,15,16] (particularly in the case of cerebral hematomas), for secondary lesions the aim is to promptly recognize or even prevent the harmful effects of the cellular damage pathways in this phase.

The primary lesion includes two types of damage: focal and diffuse. Skandsen et al. [17] state that diffuse axonal injury (DAI) is detected in about 70% of TBI cases and a combination of DAI and contusions or hematomas is found in 50%.

Focal damage is represented by cerebral contusions, which are usually caused by a direct collision. They are frequently located at the areas that are most exposed to trauma, such as basal frontal and temporal areas.

Epidural, subdural, subarachnoid or intracranial hematomas frequently occur following the injury, with subsequent vascular impairment and the onset of necrosis.

Additionally, contrecoup injuries [18] can cause secondary contusions in the tissues opposite the site of the collision.

Smith et al. [19] indicate rapid acceleration/deceleration as the main actor causing DAI. These phenomena involve violent traction of the brain tissue with consequent neuronal damage on a vascular basis followed by cerebral edema, ischemic damage and subsequent neuronal loss.

As far as the diagnosis of primary lesions is concerned, the gold standard remains radiologic exams. Specifically, Magnetic Resonance Imaging (MRI) is more sensitive than Computed Tomography (CT) in detecting DAI [20]. However, in cases of moderate to severe TBI, the first and most appropriate exam is Non-Contrast Computed Tomography (NCCT) [21].

The physio-pathogenesis of secondary injuries is related to biochemical and cellular mechanisms that start at the beginning of the initial trauma. They can progress for hours (sometimes days) and are characterized by cellular apoptosis, excitotoxicity, axon degeneration, mitochondrial dysfunction, oxidative stress, lipid peroxidation and neuroinflammation [22].

In response to brain injury, cerebral arterioles dilate, giving rise to an abnormal vascular response and reducing vessel wall oxygen consumption, followed by the production of ROS, such as superoxide anion radicals, hydrogen peroxide and hydroxyl radicals. Usually, scavenging systems keep their concentrations very low in these tissues. In case of their accumulation, an irreversible injury can be produced by lipid peroxidation and the oxidation of intracellular proteins and nucleic acids.

In addition, post-TBI tissues are characterized by a high release of excitatory amino acids in extracellular space and cerebrospinal fluid, such as glutamate and aspartate. The hyperactivation of N-methyl-d-aspartate (NMDA) receptors, induced by excessive levels of glutamate, produces ROS and nitric oxide, intensifying secondary cell injury [12].

Neuronal and oligodendrocytes apoptosis represent other mechanisms involved in secondary brain injury. The interaction of various neurochemical, cellular and molecular pathways (e.g., ERK, p38 MAPK, JAK/STAT) lead to the activation of cysteine proteases such as caspase and calpain. Caspase-dependent apoptotic cell death can be generated by the extrinsic death receptor pathway or the intrinsic mitochondrial pathway, while caspase-independent apoptosis is generated by the calpains, which are activated by the release of mitochondrial proteins through proteolysis of cytoskeletal proteins [12].

Mitochondrial dysfunction also contributes to metabolic deregulations and consequent cell death. Ca^2+^ intracellular sequestration and the excessive entry of ions into mitochondria produce ROS and depolarization of the mitochondrial membrane with the release in cytosol of cytochrome C and apoptosis-inducing factor (AIF) [12].

In addition to the aforementioned mechanisms, a potent neuronal inflammatory response takes place. Glial cells activation releases proinflammatory cytokines and chemokines, upregulates endothelial adhesion molecules and stimulates the complement system. Moreover, these events lead to the activation of leukocytes and to the perpetuation of intracranial inflammation and release of cytotoxic proteases [23].

One of the main players in the regulation of some of the above processes appears to be the transcription factor FOXO.

The FOX transcription factor family includes more than 100 proteins, divided into subfamilies. FOXO1, FOXO3a, FOXO4 and FOXO6 [24] belong to subfamily O (FOXOs). They are widely studied because they not only control physiological cellular ageing [25], but they are also implicated in the regulatory mechanisms of many pathologies, including cancer [26,27], cardiovascular diseases [28], diabetes [29] and neurodegenerative diseases [30]. Xin et al. [31] have suggested that FOXOs could be regulators of ageing and longevity, while Akhter et al. [32] have demonstrated that FOXOs are implicated in the pathogenesis of Alzheimer’s disease.

Some recent studies have shown that this class of proteins is implicated in the mechanisms of cell damage in brain tissue affected by TBI [10,11].

In particular, FOXO3a regulates cellular homeostasis, and it is widely expressed in the brain (hippocampus, cerebral cortex and cerebellum) and involved in cellular stress responses, such as the elimination of ROS [33,34], cellular ageing [35], cell cycle regulation [36] and DNA repair [37]. Moreover, it seems to be involved in autophagy and apoptosis [38].

**Figure 3:**
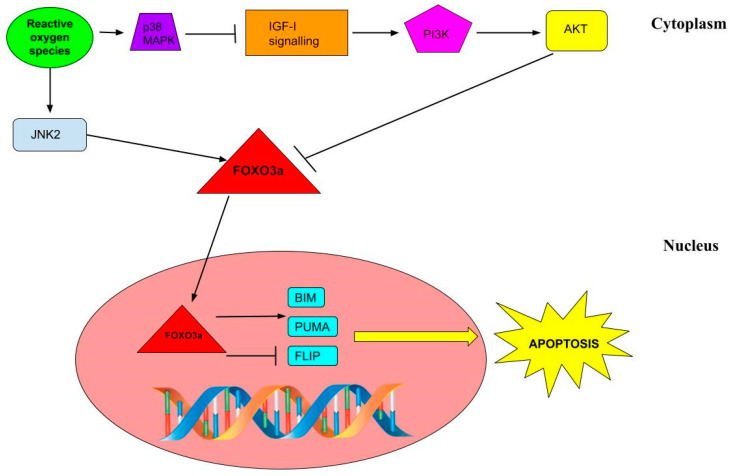
This figure shows the apoptosis pathways in which FOXO3a is involved.

As shown in Figure 3, FOXO3a expression can be inhibited by the phosphorylation of other proteins, such as AKT. On the other hand, it can be activated by the phosphorylation of other factors, such as AMP-activated protein kinase (AMPK) or JNK2. In turn, AKT is activated in the PI3K signaling pathway.

Specifically, in neurons, ROS-induced oxidative stress (e.g., H_2_O_2_) upregulates FOXO3a expression via a dual-signaling pathway. Indeed, on the one hand, it induces the inhibition of AKT, and on the other hand, it activates JNK2. AKT inhibition is crucial for FOXO3a expression. In fact, JNK2 cannot stimulate FOXO3a if AKT is still active [8].

Once activated, FOXO3a acts in the nucleus by upregulating pro-apoptotic genes, such as BIM [39] and PUMA [40], or by downregulating anti-apoptotic genes, such as FLIP [41]. In both cases, it promotes cell death.

In post-TBI tissue, FOXO3a promotes cellular apoptosis, neuroinflammation and the response to oxidative stress.

Regarding this topic, Liu XL. et al. [11] first studied the expression of FOXO1, FOXO3a and FOXO4 in the human brain and, experimentally, in the mouse brain, performing Western blot analysis and immunofluorescence staining. Their results indicate substantial expression of FOXOs in post-TBI brain tissue.

Liqian S. et al. [10], carrying out IHC studies in mice, came to a similar conclusion as Liu XL et al. and also detected a correlation between the level of FOXO3a expression and the degree of severity of the TBI.

Moreover, the aforementioned authors [10,11] observed that FOXO3a expression is time-dependent post-TBI, with a peak at 24 h.

Returning to the present work—the first IHC study conducted on human cerebral samples—the obtained results confirmed that there is a correlation between TBI and FOXO3a expression. As a matter of fact, this factor was absent in the samples of the control cases (subjects who did not undergo TBI) and it was expressed in the samples taken from TBI cases.

Furthermore, it was noted that as the survival time of the subject increased—with the maximum being 6 h—the FOXO3a expression was greater.

The results are, therefore, consistent with those of other studies.

Because of these findings, it is worth asking which clinical implications FOXO3a could have, as a possible pharmacological target.

Since in TBI cases the primary injuries generally involve clinically irreversible damage, current treatment regimens aim to stabilize the injury site and prevent further secondary damage from developing.

The mechanisms that come into action in secondary injuries are responsible for the further worsening of neurological conditions. Therefore, it is conceivable that modulating these regulatory pathways by inhibiting the expression of factors such as FOXO3a would reduce neuronal and glial cell loss. This would limit the damage, other than the consequent persistent inflammatory response, excitotoxicity, oxidative stress and apoptotic cell death, which are typically associated with these damage mechanisms [22].

Liqian Sun et al. [10] found that the knockdown of FOXO3a by siRNA silencing ensures neuroprotective effects by reducing neurobehavioral dysfunctions and therefore recognized the value of FOXO3a as an important potential therapeutic target for the treatment of TBI.

## 4. Materials and Methods

### 4.1. Study Group Selection and Sample Collection

The autopsy databases of the Legal Medicine and Forensic Institutes of the “Sapienza” University of Roma and University of Pisa were retrospectively reviewed. After analyzing the autopsy reports and the information gathered from the police investigation, 15 cases of TBI deaths were selected. The resulting study group was composed of 4 women and 11 men, with a mean age of 43.1 years. Among these 15 cases, in 8 cases death occurred instantly, and in the remaining 7 cases, death occurred within 6 h. Inclusion criteria for the study group were cause of death related to TBI, injury event occurred within the preceding 24 h and no history of chronic disease.

As a control group, another 15 cases (8 women, 7 men, mean age of 49.3 years) were chosen among non-traumatic brain deaths: 5 spontaneous cerebral aneurysm rupture cases and 10 acute cardio-circulatory arrest cases.

Decomposed bodies or those with initial signs of putrefaction were excluded from both groups.

Autoptic investigations were performed within 36 h after death. Cerebral cortex samples were collected at traumatic injury sites and where the injury was most present.

### 4.2. Histological and Immunohistochemical Analysis

A routine microscopic histopathological study was performed using hematoxylin–eosin (H&E) staining. In addition, an IHC investigation of cerebral samples was performed.

Samples 8 cm^2^ in size from each case were fixed in 10% buffered formalin, and then washed with phosphate-buffered saline (PBS), and subsequent dehydration was carried out using a graded alcohol series. After dehydration, samples were cleared in xylene and embedded in paraffin. Sections measuring 4μm were mounted on slides and covered with 3-amminopropyltriethoxysilane (Fluka, Buchs, Switzerland).

To evaluate FOXO3a expression, anti-FOXO3a antibodies (GTX100277) were utilized. They recognize both the phosphorylated and dephosphorylated forms of FOXO3a. Antigen retrieval was carried out using EDTA buffer in a pressure steamer at 100 °C for 90 min. Slides were stained on an automated immunostainer (Dako Cytomation, Glostrup, Denmark) using a polyclonal anti-FOXO3a antibody (GeneTex cat. No. GTX100277 FOXO3a Ab (C3), C-term (knockout (KO)-validated). Tryptase: 5 min Proteolytic Enzyme (Dako, Copenhagen, Denmark) was used under the following conditions: 20 °C, 120 min, 20 °C, 1:1000, and CD 15: (DAKO, Copenhagen, Denmark) was boiled in 0.25 mM EDTA buffer; 120 min, 20 °C 1:50).

**Figure 4:**
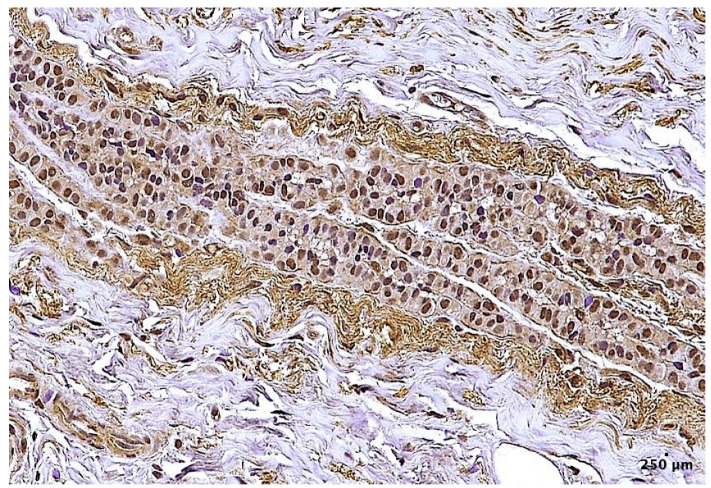
FOXO3a positivity in breast cancer. FOXO3a is localized both in the cytoplasm and nucleus (100×). The scale bar showed 250 μm in distance.

Before staining the study group’s samples, anti-FOXO3a antibodies (GTX100277) were tested on breast cancer samples, as other authors have already demonstrated FOXO3a is positive and could have a nuclear and cytoplasmatic localization in such tissues. In Figure 4, the breast-cancer-positive control is shown.

### 4.3. Quantitative Analysis

For quantitative analysis, in each IHC section, 20 observations were made in different fields/slides at 100-fold magnification. The samples were also examined under a confocal microscope, and a three-dimensional reconstruction was performed (True Confocal Scanner, Leica TCS SPE, Cambridge, UK).

The staining intensity was evaluated using a semi-quantitative scoring scale. A semi-quantitative blind evaluation of the IHC findings was performed by two different investigators (AM and VF). All measurements were carried out at the same image magnification (×10), and the gradation of the IHC reaction was used with a scale from 0 to +4. The IHC positivity score was defined as: 0, nuclear and/or cytoplasm staining absent; 1, nuclear and/or cytoplasmic staining, 25%; 2, nuclear and/or cytoplasmic staining, 50%; 3, nuclear and/or cytoplasmic staining, 75%; 4, nuclear and/or cytoplasm staining, 100%. The grade was based on the maximum positivity of FOXO3a noted. The evaluations were carried out separately for each sample, using a double-blind method. In cases of divergent scoring, a third observer (ET) decided the final score.

### 4.4. Statistical Analysis

Semi-quantitative evaluation of the IHC findings and gradation of the IHC reaction were described with an ordinal scale. The median values were then reported. Analysis of variance for the non-parametric data was performed using a Kruskal–Wallis test. When differences were found to be significant, analysis between the unmatched groups was elucidated with a Dunn’s multiple comparison post hoc test. The significance level was set to 5% (SPSS ver. 16.01 for Windows—SPSS Inc., Chicago, IL, USA).

### 4.5. Abbreviations

Traumatic brain injury (TBI); Centers for Disease Control and Prevention (CDC); Immunohistochemical (IHC); hematoxylin–eosin (H&E); Forkhead Box class O (FOXOs); phosphate-buffered saline (PBS); diffuse axonal injury (DAI); Magnetic Resonance Imaging (MRI); Computed Tomography (CT); Non-Contrast Computed Tomography (NCCT); N-methyl-d-aspartate (NMDA); apoptosis-inducing factor (AIF); AMP-activated protein kinase (AMPK).

## 5. Conclusions

This work is the first IHC study that illustrates the expression of FOXO3a in human brain samples of post-TBI cases and underlines the importance of this transcription factor in secondary post-TBI injuries. As a matter of fact, FOXO3a plays a crucial role in the regulation of neuronal autophagy and apoptosis and in post-TBI neuronal dysfunctions.

Thanks to the results of this study, which are in line with the data present in the literature, it can be understood that FOXO3a is clearly expressed in encephalic tissues that suffered from TBI.

Furthermore, the longer the time between trauma and death (survival time), the greater the nuclear/cytoplasmic accumulation of FOXO3a in neuronal cells.

The small number of cases that we studied represents a limitation for our preliminary study, but we plan to increase the number of cases analyzed in the future.

However, it is important to highlight that this study opens up new perspectives in the post-mortem diagnosis of TBI, introducing a new, promising diagnostic tool.

Additionally, by posing several questions about the physiopathological role of FOXO3a, it aims to stimulate further studies on the topic using additional laboratory techniques.

In conclusion, our results allow us to believe that our technique may represent a promising and dependable means of conducting a post-mortem diagnosis of TBI [42]. Moreover, the study shows the important role of FOXO3a in neuronal autophagy and apoptosis regulation and suggests FOXO3a as a possible potential pharmacological target to prevent the onset of secondary injuries and to improve the outcome of patients who have suffered from TBI, in terms of reducing neurological deficits [43,44,45,46,47].

## Figures and Tables

**Table 1:** This table shows the results of the semi-quantitative analysis of the IHC reaction in the cerebral section of the 15 cases of TBI. Qualitative and statistical differences were seen concerning the time of death.

Case Number	Sex	Staining Intensity	Time of Death
1	M	2	Immediately
2	F	1	Immediately
3	M	3	2 h
4	M	2	Immediately
5	M	3	3.5 h
6	M	1	Immediately
7	F	4	6 h
8	F	3	Immediately
9	F	4	6 h
10	M	2	Immediately
11	M	4	5 h
12	M	2	1 h
13	M	2	Immediately
14	M	4	4 h
15	M	1	Immediately

## Data Availability

The data presented in this study are available in Table 1.
